# How to Form Good Habits? A Longitudinal Field Study on the Role of Self-Control in Habit Formation

**DOI:** 10.3389/fpsyg.2020.00560

**Published:** 2020-03-27

**Authors:** Anouk van der Weiden, Jeroen Benjamins, Marleen Gillebaart, Jan Fekke Ybema, Denise de Ridder

**Affiliations:** ^1^Department of Social Health and Organizational Psychology, Utrecht University, Utrecht, Netherlands; ^2^Department of Social Economic and Organizational Psychology, Leiden University, Leiden, Netherlands; ^3^Department of Experimental Psychology, Utrecht University, Utrecht, Netherlands

**Keywords:** habit formation, behavior performance, trait self-control, longitudinal app study, community sample

## Abstract

When striving for long-term goals (e.g., healthy eating, saving money, reducing energy consumption, or maintaining interpersonal relationships), people often get in conflict with their short-term goals (e.g., enjoying tempting snacks, purchasing must-haves, getting warm, or watching YouTube video’s). Previous research suggests that people who are successful in controlling their behavior in line with their long-term goals rely on effortless strategies, such as good habits. In the present study, we aimed to track how self-control capacity affects the development of good habits in real life over a period of 90 days. Results indicated that habit formation increased substantially over the course of three months, especially for participants who consistently performed the desired behavior during this time. Contrary to our expectations, however, self-control capacity did not seem to affect the habit formation process. Directions for future research on self-control and other potential moderators in the formation of good habits are discussed.

## Introduction

Sometimes people find themselves mindlessly watching TV while they had the intention to be more physically active; eating sweets while they wanted to eat more healthily; or lashing out at others while they wanted to be more patient or open-minded. Sounds familiar? Although people may often be able to control themselves in order to attain long-term goals such as healthy living or maintaining satisfactory relationships, there are also many instances in which they are unable or unwilling to exert self-control (e.g., when temptations are strong or when tired; e.g., Muraven and Slessareva, 2003; Baumeister et al., 2007; Hofmann et al., 2010). Also, some people are less successful in controlling their behaviors than others (Schmeichel and Zell, 2007). In these cases, people often revert to effortless, habitual behavior (Ouellette and Wood, 1998; Webb and Sheeran, 2006; Neal et al., 2013) – often bad habits. This reliance on habits may, however, also be used to peoples’ advantage if they manage to form good habits that are in line with their long-term goals. Indeed, recent research suggests that people who are successful in controlling their behavior, more effortlessly rely on good habits (Adriaanse et al., 2014; Gillebaart and de Ridder, 2015). But how are good habits formed?

Research on habit formation has shown that behavior is likely to become habitual when it is frequently and consistently performed in the same context (e.g., Ouellette and Wood, 1998). For example, when one frequently and consistently eats vegetables for lunch, at some point eating vegetables for lunch will become a habit. This is because the frequent co-occurrence of context and behavior instigates an association that may guide future behavior (e.g., Aarts and Dijksterhuis, 2000; Neal et al., 2012). Specifically, when encountering a context (e.g., having lunch) that is associated with a certain behavior (e.g., eating vegetables), this context will automatically trigger this associated behavior. Hence, once a good habit is formed, it is rather effortless to perform desired behavior. However, the process of habit formation itself may vary in the amount of effort needed – although some people manage to form certain habits as quickly as 18 days, others need as much as half a year (Lally et al., 2010). This raises the question how exactly do habits form over time?

Although research on habit formation is still in its infancy, recent studies have uncovered some of the mechanisms that underlie the habit formation process. One of the main findings is that the habit formation process within individuals unfolds asymptotically (Lally et al., 2010; Fournier et al., 2017). That is, habit strength increases steeply at first, and then levels off. In addition, studies that studied habit formation on the group level (i.e., averaging over participants) have provided insight into the processes that facilitate such increases in habit strength. Specifically, the frequency and consistency with which the desired behavior is performed, the inherently rewarding nature of the behavior, a comfortable environment (e.g., no threats or obstacles), and easy rather than difficult behaviors have been shown to facilitate the process of habit formation (Kaushal and Rhodes, 2015; Fournier et al., 2017).

Besides these factors, there are still many others unexplored that may explain the variation in the time it takes people to form a habit. One such likely candidate is self-control capacity. That is, habit formation crucially depends on the repeated performance of behavior that is in line with one’s long-term goal. The initiation of such new behavior, as well as the inhibition of acting upon short-term temptations is likely to require effortful self-control, especially in the early stages of habit formation. Indeed a study among teenagers indicates that those who initially had higher self-control capacity reported having stronger meditation habits after three months of meditation sessions (Galla and Duckworth, 2015, Study 5). Other studies revealed that habit strength mediates the effect of self-control strength and behavior. Specifically, self-control was related to increased habit strength, which was in turn related to increased exercise behavior (Gillebaart and Adriaanse, 2017) and decreased snack intake (Adriaanse et al., 2014). However, although these studies have indicated that self-control is related to habit strength, they do not provide insight in the role of self-control capacity in the initial stages of habit formation.

The current study was a first attempt to track how self-control capacity affects the development of good habits in daily life over a relatively long period of time. We expected both repeated goal-congruent behavior performance and self-control capacity to facilitate the formation of good habits. Possibly, self-control capacity may affect habit formation via increased behavior performance (as the initiation of new behavior and inhibition of conflicting behavior requires self-control at first). To test these hypotheses, we recruited people who wanted to form a good habit in the domain of health behavior (eating fruit or vegetables, exercising, or drinking water), interpersonal relationships (making more contact with others, being more patient or open-minded, or having more attention for others), personal finance (saving money), or environmental-friendly behavior (recycling). Over the course of three months, we then measured their goal-congruent behavior performance, self-control capacity, and habit strength to examine how self-control related to behavior performance and habit strength over time.

## Methods

### Participants and Design

A community sample was recruited via the population register of the city of Utrecht in the Netherlands as well as social media and the alumni register of Utrecht University. Anyone between the age of 18 and 65 who possessed a smartphone was eligible (we could provide a limited number of participants with a smartphone for the duration of the study if they did not possess one, *N* = 5). All participants indicated they wanted to form a habit in the health, sustainability, interpersonal, or financial domain.

The within-subjects design consisted of a pre-measurement administered in groups of 2–13 participants at a university location,^1^ followed by a three-month interval of daily measures administered through an in-house developed mobile application, and after 90–110 days, a post-measurement (again in group sessions at a university location). In total, 180 people participated in the pre-measurement, of whom 90 participated in the post-measurement. Participants took part in the daily measures over a range of 17–110 days (*M* = 77.0, SD = 26.7). During this time period, the number of bi-weekly self-control assessments ranged from 1 to 10 (*M* = 6.5, SD = 2.3), which were alternated with bi-weekly habit strength assessments, of which the number ranged from 2 to 9 (*M* = 5.7, SD = 2.0). In total, 146 participants (118 women; *M*_age_ = 31.9; SD_age_ = 12.7; range 18–61 years) who completed at least one follow-up assessment of habit strength were included in the analyses. More than half of them (65.8%) were community residents (including alumni) and the remainder (34.2%) were bachelor students. Based on participants’ postal code (which is indicative of education, income, and work status; Netherlands Institute for Social Research), we inferred their socio-economic status. About 10.3% of the participants lived in underprivileged neighborhoods, 61.0% lived in middle class neighborhoods, and 26.0% came from privileged neighborhoods (postal code data was missing for 4 participants). Participants’ initial level of habit strength was moderate (*M* = 3.1, SD = 1.1).

### Procedure and Materials

#### Registration

Those who were interested in participating received an information letter via e-mail, containing a link to register for the study with a unique participation code. In the registration form, participants were reminded of the terms and conditions (i.e., voluntary nature of participation, ability to withdraw without explanation, etc.), after which they were required to give their consent for participating in the study. Participants could then schedule an appointment for the pre-measurement.

#### Pre-measurement

Participants came to the university for a pre-measurement as part of a larger longitudinal prospective study on trait self-control (i.e., to see whether self-control could be trained by daily performance of a behavior that requires self-control – which indeed seemed to be the case; de Ridder et al., 2019). As such, the different measurements (pre-, app-, and post-) also included measures that were not of interest for the current study.^2^

##### Goal setting

**FIGURE 1:**
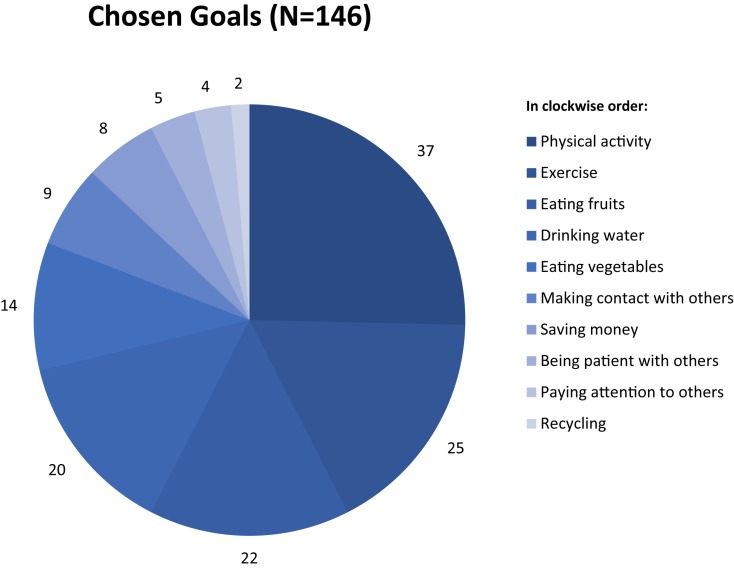
Overview of the number of participants selecting each behavior. Please note that exercise (“sporten” in Dutch) and physical activity (“bewegen” in Dutch) refers to different types of behaviors. Whereas exercise is typically associated with certain rules and competitiveness, but most of all with high intensity (e.g., playing football, cross fit, running), physical activity refers to more casual and less intense behaviors (e.g., walking or biking, gardening, household chores).

At the start of the study, participants selected a specific behavior they wanted to turn into a habit over the course of the study. Choices covered health, interpersonal, financial, and ecological behaviors (e.g., eating fruit, being patient, saving money, recycling). Depending on the type of behavior chosen, participants could then choose from three to seven contexts for behavioral practice (e.g., eating fruit when having breakfast, being patient when talking to someone,^3^ saving money when in the supermarket, or recycling when tidying up). As such, participants could choose which habit they wanted to form based on 60 preset combinations of behaviors and contexts. See Figure 1 for an overview of which behaviors were selected by the participants. It was emphasized that the selected behavior needed to be personally relevant to them, had to be a behavior they did not regularly perform yet, and had to be feasible for them to perform on a daily basis. After selecting a behavior and context, participants had to specify for themselves what this behavior entailed (e.g., when they chose exercise as their goal, it was explained that a ten minute routine at home was more feasible on a daily basis than an hour at the gym). As such, participants were intrinsically motivated and there was room for forming a new habit.

##### App instructions

For the purpose of this study, we developed a mobile app (which ran on iOS and android) to assess self-control capacity and habit strength on a regular basis. At the end of the pre-measurement, participants were instructed to install and use this app for daily tests and questionnaires. Participants were also informed that they would receive a reminder every morning via the mobile app.

#### App Measurements

##### Habit strength

Habit strength was assessed bi-weekly with the Self-Report Habit Index (Verplanken and Orbell, 2003), which consists of 12 statements (e.g., ‘[self-chosen behavior (e.g., eating fruit)] is something I do …frequently; …automatically; …without thinking)’. For each statement, participants indicated to what extent they felt the statement applied to them on a scale from 1 (completely disagree) to 7 (completely agree). The scale proved reliable with a Cronbach’s alpha of.94.^4^

##### Goal-congruent behavior performance

On a daily basis, participants indicated (dichotomously) whether or not they had performed the self-chosen behavior that day, and whether they performed this behavior in their self-chosen context.^5^

##### Self-control capacity

Self-control capacity was assessed bi-weekly with the Brief Self-Control Scale (Tangney et al., 2004), which consists of 13 statements (e.g., “I am good at resisting temptation” or “People would say I have iron self-discipline”). For each statement, participants indicated to what extent they felt the statement applied to them on a scale from 1 (not at all) to 5 (very much). The scale proved reliable with a Cronbach’s alpha of 0.79.

## Data Analysis

### Habit Formation Over Time – Individual Level Analysis

**FIGURE 2:**
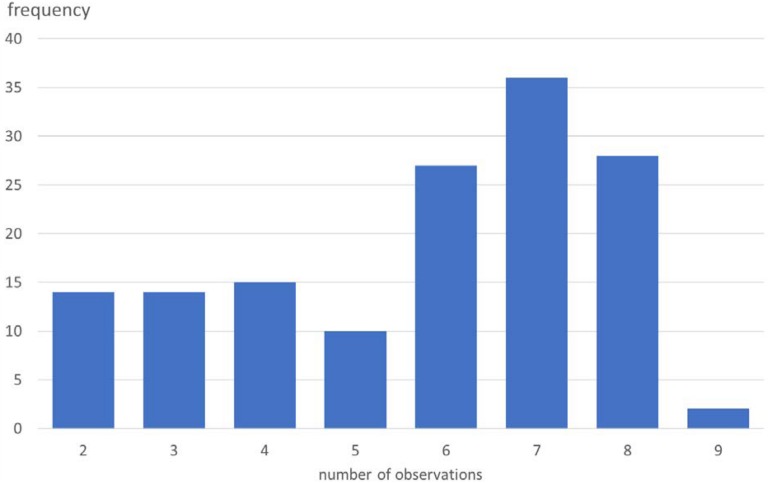
Number of observations for habit strength (total *N* = 836) plotted against the number of participants (*N* = 146).

First, following Lally et al. (2010) approach, we attempted to fit an asymptotic curve to individual participants’ habit strength scores over time (by means of a Least Squares Curve Fit algorithm in Matlab), to then see whether we could predict the individual (rate of) change in habit strength as a function of goal-congruent behavior performance and self-control capacity. However, the individual patterns fluctuated too much (possibly because bi-weekly measurements were too infrequent; *M* = 5.73, SD = 1.99, range = 2–9 observations per participant; see Figure 2 for the number of observations plotted against the number of participants^6^), and curve fitting could only be achieved for 4.11% of our participants (see results under point 2, Supplementary Material). As an alternative, we also tried fitting a less constrained power curve (*y* = ax^b^), with even less success (2.4%). We therefore decided to analyze the data on the group level instead.

### Habit Formation Over Time – Group Level Analysis

We examined the data in SPSS 24 with the Linear Mixed Models, using Maximum Likelihood estimation. In the first analysis, we carried out a growth curve modeling for habit formation, in which a random intercept, and fixed effects of a linear and a quadratic time trend were estimated. In addition, the random slopes of the linear and quadratic trend were tested to allow for individual differences in the growth curve. In a second analysis we tested whether habit formation was influenced by self-control capacity and the performance of the behavior. In Model 1, the random intercept was included to determine the intraclass correlation (ICC) of habit strength as an indicator of the variance at person level. In Model 2, lagged habit strength (i.e., habit strength at the previous measurement) was entered to analyze habit formation. Because we controlled for lagged habit strength, the linear and quadratic trend were not included in this analysis. In Model 3, self-control capacity at the previous bi-weekly measurement of self-control and daily practice of the chosen behavior (measured by the proportion of daily app-measurements in which the chosen behavior was performed during the interval between the previous and the current habit assessment) was entered, as well as a number of control variables, i.e., the measurement number of bi-weekly habit assessment, the length of the interval since the previous habit assessments, and the number of daily behavioral assessments.

## Results

### Habit Formation Over Time

**FIGURE 3:**
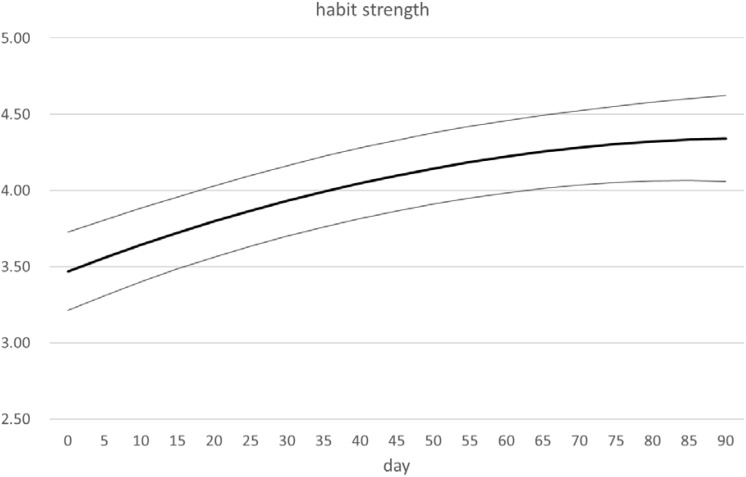
Habit strength fitted as a function time, with 95% confidence bands.

We first examined whether habit strength increased over time. Figure 3 shows a significant increase of about 0.8 SD (a large effect size according to Cohen, 1992) in habit strength over a period of 110 days with a stronger increase in beginning of the study period, leveling off at the end. Both the linear trend (*t* = 15.30, *p* < 0.001) and the quadratic trend (*t* = −3.39, *p* < 0.001) were significant. Adding the random slopes for the linear (Wald *Z* = 5.37, *p* < 0.001) and quadratic (Wald *Z* = 2.40, *p* < 0.05) improved the fit of the model, showing that habit formation differed over participants.

### Effects of Goal-Congruent Behavior Performance and Self-Control Capacity on Habit Formation

Table 1 shows the results of a hierarchical multilevel analysis of habit formation. As can be seen in Model 2, habit strength is rather stable and strongly predicted by lagged habit strength at the previous measurement of habit. Nevertheless, entering lagged self-control capacity and goal-congruent behavior performance in the time period during both habit strength measurements further increased the fit of the model. Self-control capacity did not contribute to higher habit strength^7^. However, participants who carried out the self-chosen behavior more consistently (higher proportion of goal-congruent behavior performance^8^), showed stronger increases in habit strength. In line with the trend in habit formation shown before, the time of measurement (i.e., the umpteenth time) had a small negative influence on habit strength increase. This is in line with the lower increase in habit strength later on during the study period.

**TABLE 1:** The multilevel regression of habit strength.

Predictors	Model 1	Model 2	Model 3
Intercept	4.07***	4.12***	4.13***
Lagged habit strength		0.87***	0.85***
Lagged self-control			0.01
Time of measurement			−0.03*
Days between measurements			0.00
Number of app-measurements			0.00
Proportion behavior carried out			0.47***
Fit(−2 log L)	1445.05***	1,095.91***	1067.58***
Δ fit		349.14***	28.33***
df		1	5
**Variance**			
Random intercept (person level)	1.16***	0.00	0.00
Residual (day level)	0.30***	0.32***	0.31***
ICC	0.80		
Explained variance		78%	79%

* *p* < 0.05; *** *p* < 0.001.

## Discussion

People often struggle in the pursuit of their long-term goals. As good habits may help people in this pursuit, we set out to gain more insight in how good habits are formed in daily life. We specifically focused on goal-congruent behavior performance and self-control capacity as potential facilitators of habit formation. We were able to test our hypotheses in a diverse and highly committed sample. Results showed a large increase in habit strength over the course of three months, which was strongest for participants who consistently performed the self-chosen goal-congruent behavior during this time. Contrary to our expectations and previous findings by Galla and Duckworth (2015), however, we did not find support for self-control capacity as a predictor of the habit formation process.

One reason why self-control capacity may not have facilitated habit formation, could be that participants experienced little conflict between their long-term goal and an immediately gratifying alternative. In contrast to well-controlled lab experiments where participants are simultaneously confronted with goal-congruent stimuli (e.g., broccoli) and conflicting temptations (e.g., apple pie), such temptations may not always be present when the opportunity presents itself to perform goal-congruent behavior in real life. If so, the reason that participants did not yet regularly perform the desired behavior before participating in the study, may not have been because they were unable to control their behavior in the presence of temptations. Alternatively, in the absence of temptation, participants may have had difficulty monitoring their behavior and identifying opportunities for goal pursuit. In the current study monitoring was facilitated by specifying a specific context for goal pursuit and registering their behavior daily via the smartphone application, which may have facilitated goal-congruent behavior performance, and hence, habit formation. Indeed, monitoring has been proven to be very effective in goal progress and attainment (see Harkin et al., 2016 for meta-analyses; Michie et al., 2009). Future research could extend the current findings by assessing how often people run into temptations during long-term goal pursuit and whether its impact on the habit formation process is modulated by self-control capacity. Also, future research could investigate whether habit formation can be facilitated even more by frequent monitoring at regular intervals during the day.

Another reason why self-control may not have affected habit formation, is because our instructions to participants may have created an association between the specific, self-chosen behavior and a specific context. Research has shown that if people form specific “if…, then…” plans (also referred to as implementation intentions), in which a specific behavior is linked to a specific context (e.g., if I open the fridge, then I will grab the cherry tomatoes), this will automatically trigger the specific behavior upon encounter of the specific context (Gollwitzer, 1999; Webb and Sheeran, 2007). As such, habit strength – or rather, behavioral automaticity – should increase instantly and self-control is no longer required. Although we did not ask our participants to form implementation intentions, our request to select a specific context in which to perform the specific self-chosen behavior may have resulted in cue-behavior associations that facilitate effortless behavior performance. However, our data as well as the data of Lally and colleagues (Lally et al., 2010; in which implementation intentions were actually formed) do not seem to support this line of reasoning. Even if cue-behavior associations were formed, they did not result in instant increases in habit strength, as habit formation unfolded gradually over the course of several months, leaving room for self-control capacity to influence the habit formation process. It would be interesting, though, to further investigate the role of self-control capacity in the presence versus absence of cue-behavior associations in an experimental field study.

Yet another reason for not finding an effect of self-control on the habit formation process may be that we focused on trait rather than state self-control. Although trait self-control did increase over time (see de Ridder et al., 2019; and hence, may have benefited the habit formation process), trait self-control is a relatively stable factor. Future research should assess within-individual fluctuations of state self-control in the habit formation process – preferably also fitting habit formation on the individual level. Our findings suggest that more data points are required for such analyses.

In line with previous research (Lally et al., 2010; Fournier et al., 2017), the current (aggregated) data provided support for the asymptotic contribution of repeated goal-congruent behavior performance to the formation of habit. Unfortunately, we were unable to show this trend on the individual level, due to the bi-weekly assessment of habit strength. Hence, future studies would benefit from more frequent assessments. These studies may also want to test further moderators of habit formation, e.g., what type of contextual cues may be the best triggers for behavior, the role of motivation, and how the formation of good habits affect the bad habits they aim to substitute (see also Gardner and Lally, 2018).

Beside the strengths of our study (a diverse and highly committed sample), it is important to note that the self-report measurement of habit strength may have been subject to biases. Although the SRHI is commonly used and well validated (Verplanken and Orbell, 2003; Gardner et al., 2011), it would be even more compelling if the current findings could be corroborated by more implicit measures of habit strength, such as a lexical decision task (Meyer et al., 1972). In the current study, we have attempted to measure habit strength by means of a lexical decision task in the mobile app. However, the mobile app measurements were not sensitive enough to detect any effects (see point 5, Supplementary Material). Future research may instead opt for online computer measurements.

To conclude, our study was the first to track the role of self-control capacity in the habit formation process in a longitudinal field experiment. Although we did not find evidence for self-control as a facilitator of habit formation, the current findings do offer new directions for future research on self-control and other potential moderators in the formation of good habits.

## Data Availability Statement

The datasets generated for this study are available on request to the corresponding author.

## Ethics Statement

The studies involving human participants were reviewed and approved by The Faculty Ethics Review Board – Faculty of Social and Behavioral Sciences at Utrecht University. The patients/participants provided their written informed consent to participate in this study.

## Author Contributions

AW, JB, MG, and DR developed the theory and study design. AW carried out the experiment and data preparations, and took the lead in writing of the manuscript. AW and JB performed the individual-level analyses. JY performed the group-level analyses. All authors provided critical feedback and helped to shape the analyses and manuscript.

## Conflict of Interest

The authors declare that the research was conducted in the absence of any commercial or financial relationships that could be construed as a potential conflict of interest.

## Footnotes

1 The group administration served to allow more participants to start around the same time, i.e., to minimize seasonal influences (e.g., new year’s resolutions). We minimized the degree to which participants influenced each other by stressing the importance of independent answers and reactions, as well as the importance of being silent during the measurements. Also, one or two researchers were always present to monitor participants and answer questions.

2 In the pre-measurement we measured explicit habit strength (with the Self-Report Habit Index; Verplanken and Orbell, 2003) and implicit habit strength (by means of a Lexical Decision Task), implicit state self-control (an adapted version of the mouse-tracker task; Freeman and Ambady, 2010) and explicit self-control capacity (Brief Self-Control Scale; Tangney et al., 2004), general attributional style (General Attributional Style Questionnaire; Peterson et al., 1982), goal importance, and motivation. In the smartphone app, behavioral performance, context encounter, and attributions of failure were measured daily, while habit formation, self-control capacity, general self-efficacy (General Self-Efficacy Scale; Jerusalem and Schwarzer, 1979), and willpower beliefs (Job et al., 2010) were measured bi-weekly. Additionally, a mouse tracker task was alternated with a lexical decision task every other day to measure implicit self-control and implicit habit formation, respectively. During the post-measurement, participants completed the same tasks and questionnaires as during the pre-measurement, except that the General Attributional Style Questionnaire was replaced by an ego-depletion task.

3 The option “when talking to someone” could be further specified into “when talking to a friend/partner/parent/child/neighbor”.

4 We have also run the analyses with the SRBAI subscale, which led to the same results (see point 1, Supplementary Material).

5 For our main analysis, we looked at whether the behavior was performed or not, regardless of the context it was performed in. Analyzing whether the behavior was performed in context or not yielded similar results.

6 Participants for whom an asymptotic curve could be fitted did not differ from participants for whom an asymptotic curve could not be fitted in their number of data points [M = 6.33, SD = 2.25 vs. M = 6.6, SD = 1.42, respectively; *F*(1,116) = 0.19, *p* = 0.67] or behavioral consistency [M = 0.88, SD = 0.15 vs. M = 0.77, SD = 0.26, respectively; *F*(1,144) = 1.02, *p* = 0.31].

7 One might argue that the effect of self-control capacity on habit formation is via goal-congruent behavior performance. However, our data do not provide support for a relationship between lagged self-control and goal-congruent behavior performance (see de Ridder et al., 2019). Also, entering lagged self-control in the model first (in Model 3), and subsequently adding the other variables (in Model 4), does not reveal any relationship between self-control and habit strength (see point 4, Supplementary Material).

8 Behavioral consistency differed between the different behaviors chosen [*F*(9,136) = 3.02, *p* = 0.003, η^2^ = 0.17], such that people were most consistent in performing prosocial behaviors, whereas people were least consistent in exercising and saving money (see point 3, Supplementary Material). Adding the chosen behavior to the model did not improve the model fit [Δχ^2^ (df = 9) = 7.10 ns], nor did it change any of the reported effects.

## References

[B1] Aarts H. Dijksterhuis A. (2000). Habits as knowledge structures: automaticity in goal-directed behavior. *J. Pers. Soc. Psychol.* 78 53–63. 10.1037/0022-3514.78.1.53 10653505

[B2] Adriaanse M. A. Kroese F. M. Gillebaart M. De Ridder D. T. D. (2014). Effortless inhibition: habit mediates the relation between self-control and unhealthy snack consumption. *Front. Psychol.* 5:444. 10.3389/fpsyg.2014.00444 24904463 PMC4032877

[B3] Baumeister R. F. Vohs K. D. Tice D. M. (2007). The strength model of self-control. *Curr. Direct. Psychol. Sci.* 16 351–355. 10.1111/j.1467-8721.2007.00534.x

[B4] Cohen J. (1992). A power primer. *Psychol. Bull.* 112 155–159. 19565683 10.1037//0033-2909.112.1.155

[B5] de Ridder D. van der Weiden A. Gillebaart M. Benjamins J. Ybema J. F. (2019). Just do it: engaging in self-control on a daily basis improves the capacity for self-control. *Motiv. Sci*. 10.1037/mot0000158

[B6] Fournier M. d’Arripe-Longueville F. Rovere C. Easthope C. S. Schwabe L. El Methni J. (2017). Effects of circadian cortisol on the development of a health habit. *Health Psychol.* 36 1059–1064. 10.1037/hea0000510 28650196

[B7] Freeman J. B. Ambady N. (2010). MouseTracker: software for studying real-time mental processing using a computer mouse-tracking method. *Behav. Res. Methods* 42 226–241. 10.3758/BRM.42.1.226 20160302

[B8] Galla B. M. Duckworth A. L. (2015). More than resisting temptation: beneficial habits mediate the relationship between self-control and positive life outcomes. *J. Pers. Soc. Psychol.* 109 508–525. 10.1037/pspp0000026 25643222 PMC4731333

[B9] Gardner B. de Bruijn G.-J. Lally P. (2011). A systematic review and meta-analysis of applications of the self-report habit index to nutrition and physical activity behaviours. *Ann. Behav. Med.* 42 174–187. 10.1007/s12160-011-9282-0 21626256

[B10] Gardner B. Lally P. (2018). *Modelling Habit Formation and Its Determinants. In The Psychology of Habit* (Cham: Springer International Publishing), 207–229.

[B11] Gillebaart M. Adriaanse M. A. (2017). Self-control predicts exercise behavior by force of habit, a conceptual replication of Adriaanse et al. (2014). *Front. Psychol.* 8:190. 10.3389/fpsyg.2017.00190 28243217 PMC5303741

[B12] Gillebaart M. de Ridder D. T. D. (2015). Effortless self-control: a novel perspective on response conflict strategies in trait self-control. *Soc. Pers. Psychol. Compass* 9 88–99. 10.1111/spc3.12160

[B13] Gollwitzer P. M. (1999). Implementation intentions: strong effects of simple plans. *Am. Psychol.* 54 493–503. 10.1037/0003-066X.54.7.493

[B14] Harkin B. Webb T. L. Chang B. P. I. Prestwich A. Conner M. Kellar I. (2016). Does monitoring goal progress promote goal attainment? A meta-analysis of the experimental evidence. *Psychol. Bull.* 142 198–229. 10.1037/bul0000025 26479070

[B15] Hofmann W. van Koningsbruggen G. M. Stroebe W. Ramanathan S. Aarts H. (2010). As pleasure unfolds. Hedonic responses to tempting food. *Psychol. Sci.* 21 1863–1870. 10.1177/0956797610389186 21106885

[B16] Jerusalem M. Schwarzer R. (1979). The general self-efficacy scale.

[B17] Job V. Dweck C. S. Walton G. M. (2010). Ego depletion–is it all in your head? implicit theories about willpower affect self-regulation. *Psychol. Sci.* 21 1686–1693. 10.1177/0956797610384745 20876879

[B18] Kaushal N. Rhodes R. E. (2015). Exercise habit formation in new gym members: a longitudinal study. *J. Behav. Med.* 38 652–663. 10.1007/s10865-015-9640-7 25851609

[B19] Lally P. van Jaarsveld C. H. M. Potts H. W. W. Wardle J. (2010). How are habits formed: modelling habit formation in the real world. *Eur. J. Soc. Psychol.* 40 998–1009. 10.1002/ejsp.674

[B20] Meyer D. E. Schvaneveldt R. W. Ruddy M. G. (1972). “Activation of lexical memory,” in *Proceedings of the meeting in the Psychonomic Society*, St. Louis.

[B21] Michie S. Abraham C. Whittington C. McAteer J. Gupta S. (2009). Effective techniques in healthy eating and physical activity interventions: a meta-regression. *Health Psychol.* 28 690–701. 10.1037/a0016136 19916637

[B22] Muraven M. Slessareva E. (2003). Mechanisms of self-control failure: motivation and limited resources. *Pers. Soc. Psychol. Bull.* 29 894–906. 10.1177/0146167203029007008 15018677

[B23] Neal D. T. Wood W. Drolet A. (2013). How do people adhere to goals when willpower is low? The profits (and pitfalls) of strong habits. *J. Pers. Soc. Psychol.* 104 959–975. 10.1037/a0032626 23730907

[B24] Neal D. T. Wood W. Labrecque J. S. Lally P. (2012). How do habits guide behavior? Perceived and actual triggers of habits in daily life. *J. Exp. Soc. Psychol.* 48 492–498. 10.1016/j.jesp.2011.10.011

[B25] Ouellette J. Wood W. (1998). Habit and intention in everyday life: the multiple processes by which past behavior predicts future behavior. *Psychol. Bull.* 124 54–74. 10.1037/0033-2909.124.1.54

[B26] Peterson C. Semmel A. von Baeyer C. Abramson L. Y. Metalsky G. I. Seligman M. E. P. (1982). The attributional style questionnaire. *Cogn. Ther. Res.* 6 287–299. 10.1007/BF01173577

[B27] Schmeichel B. J. Zell A. (2007). Trait self-control predicts performance on behavioral tests of self-control. *J. Pers*. 75, 743–756. 10.1111/j.1467-6494.2007.00455.x 17576357

[B28] Tangney J. P. Baumeister R. F. Boone A. L. (2004). High self-control predicts good adjustment, less pathology, better grades, and interpersonal success. *J. Pers.* 72 271–324. 10.1111/j.0022-3506.2004.00263.x 15016066

[B29] Verplanken B. Orbell S. (2003). Reflections on past behavior: a self-report index of habit strength1. *J. Appl. Soc. Psychol.* 33 1313–1330. 10.1111/j.1559-1816.2003.tb01951.x

[B30] Webb T. L. Sheeran P. (2006). Does changing behavioral intentions engender behavior change? A meta-analysis of the experimental evidence. *Psychol. Bull.* 132 249–268. 10.1037/0033-2909.132.2.249 16536643

[B31] Webb T. L. Sheeran P. (2007). How do implementation intentions promote goal attainment? A test of component processes. *J. Exp. Soc. Psychol.* 43 295–302. 10.1016/J.JESP.2006.02.001

