# Supplementary material for: How to Form Good Habits? A Longitudinal Field Study on the Role of Self-Control in Habit Formation
Source: Front Psychol. 2020 Mar 27;11:560. doi: 10.3389/fpsyg.2020.00560 (PMC7135855; doi:10.3389/fpsyg.2020.00560)
Supplement: Supplementary file 1 [file Table_1.DOCX]

**Additional analyses habit formation**

1. **Behavioral Automaticity (SRBAI) Over Time – group level**

We first examined whether behavioral automaticity increased over time. Figure 2 shows a significant increase of about 0.8 SD (a large effect size according to Cohen, 1992) in behavioral automaticity over a period of 110 days with a stronger increase in beginning of the study period, levelling off at the end. Both the linear trend (t = 14.22, p < .001) and the quadratic trend (t = -2.96, p < .01) were significant. Adding the random slopes for the linear (Wald Z = 5.15, p < .001) and quadratic (Wald Z = 2.10, p < .05) improved the fit of the model, showing that habit formation differed over participants.


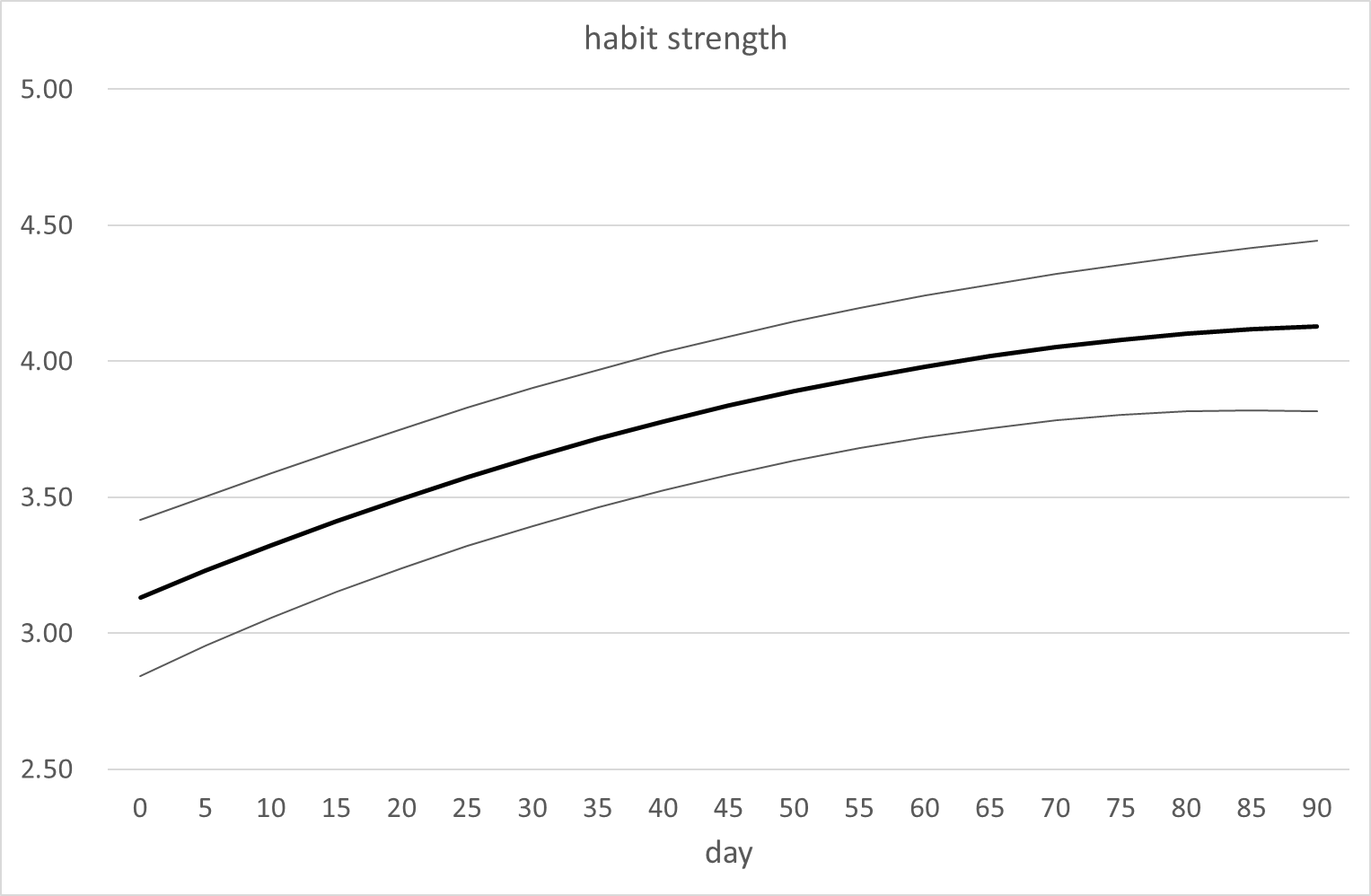


**Figure 1.** Behavioral Automaticity (SRBAI) Over Time

**Table 1.** Regression table SRBAI

| **Predictors** | **Model 1** |  | **Model 2** |  | **Model 3** |  | **Model 4** |  |
| --- | --- | --- | --- | --- | --- | --- | --- | --- |
| Intercept | 3.80 | ^***^ | 3.87 | ^***^ | 3.87 | ^***^ | 3.88 | ^***^ |
| lagged habit strength (SRBAI) |  |  | 0.85 | ^***^ | 0.85 | ^***^ | 0.83 | ^***^ |
| lagged self-control |  |  |  |  | 0.01 |  | 0.02 |  |
| measurement number |  |  |  |  |  |  | -0.03 |  |
| days between measurements |  |  |  |  |  |  | 0.01 |  |
| number of app-measurements |  |  |  |  |  |  | -0.00 |  |
| proportion behavior carried out |  |  |  |  |  |  | 0.57 | ^***^ |
| **Fit (-2 log L)** | 1670.12 | ^***^ | 1393.41 | ^***^ | 1393.38 | ^***^ | 1367.76 | ^***^ |
| Δ fit |  |  | 276.71 | ^***^ | 0.03 |  | 25.62 | ^***^ |
| Df |  |  | 1 |  | 1 |  | 4 |  |
| **Variance** |  |  |  |  |  |  |  |  |
| random intercept (person level) | 1.43 | ^***^ | 0.00 |  | 0.00 |  | 0.00 |  |
| residual (day level) | 0.44 | ^***^ | 0.51 | ^***^ | 0.51 | ^***^ | 0.49 | ^***^ |
| ICC | 0.76 |  |  |  |  |  |  |  |
| explained variance |  |  | 73% |  | 73% |  | 74% |  |
| Note: * p < .05; ** p < .01; *** p < .001. |  |  |  |  |  |  |  |  |

1. **Behavioral Automaticity (SRBAI) Over Time – individual level**

**Table 2.** Median, quartiles and minimum and maximum values of the curve parameters for those for whom the asymptotic model could be fitted (N = 21). Note that we have reported SRBAI sum scores here for comparability to Lally et al., 2010.

|  | Median | Quartiles (Q_1_ : Q_3_) | Minimum | Maximum |
| --- | --- | --- | --- | --- |
| *R*^2^ | <0.001 | <0.001 : 0.46 | <0.001 | 0.91 |
| a (asymptote) | 18.09 | 14.15 : 21.79 | 8.00 | 22.00 |
| *b* (change in habit strength/automaticity) | 2.09 | -0.03 : 6.62 | -0.11 | 15.66 |
| *c* (rate of change) | 3.35 | 0.04 : 6.06 | 0.003 | 6.42 |

1. **Behavioral consistency for the different self-chosen behaviors**

| **Self-Chosen Behavior** | **Behavioral Consistency** | |
| --- | --- | --- |
|  | Mean | SD |
| Eating fruits | .79 | .27 |
| Eating vegetables | .76 | .27 |
| Drinking water | .89 | .18 |
| Exercise | .59 | .29 |
| Physical activity | .78 | .23 |
| Making contact with others | .91 | .15 |
| Being patient with others | .90 | .14 |
| Paying attention to others | .95 | .07 |
| Saving money | .61 | .33 |
| Recycling | .90 | .14 |

1. **Lagged self-control in isolation (Model 3) or in combination with other variables (Model 4)**

**Table 3.** Regression table for habit strength (SRHI) in which lagged self-control is separately entered in Model 3.

| **Predictors** | **Model 1** |  | **Model 2** |  | **Model 3** |  | **Model 4** |  |
| --- | --- | --- | --- | --- | --- | --- | --- | --- |
| Intercept | 4.07 | ^***^ | 4.12 | ^***^ | 4.13 | ^***^ | 4.13 | ^***^ |
| lagged habit strength |  |  | 0.87 | ^***^ | 0.86 | ^***^ | 0.85 | ^***^ |
| lagged self-control |  |  |  |  | -0.00 |  | 0.01 |  |
| measurement number |  |  |  |  |  |  | -0.03 | ^*^ |
| days between measurements |  |  |  |  |  |  | 0.00 |  |
| number of app-measurements |  |  |  |  |  |  | 0.00 |  |
| proportion behavior carried out |  |  |  |  |  |  | 0.47 | ^***^ |
| **Fit (-2 log L)** | 1445.05 | ^***^ | 1,095.91 | ^***^ | 1095.89 | ^***^ | 1067.58 | ^***^ |
| Δ fit |  |  | 349.14 | ^***^ | 0.02 |  | 28.31 | ^***^ |
| Df |  |  | 1 |  | 1 |  | 4 |  |
| **Variance** |  |  |  |  |  |  |  |  |
| random intercept (person level) | 1.16 | ^***^ | 0.00 |  | 0.00 |  | 0.00 |  |
| residual (day level) | 0.30 | ^***^ | 0.32 | ^***^ | 0.32 | ^***^ | 0.31 | ^***^ |
| ICC | 0.80 |  |  |  |  |  |  |  |
| explained variance |  |  | 78% |  | 78% |  | 79% |  |
| Note: * p < .05; ** p < .01; *** p < .001. |  |  |  |  |  |  |  |  |

1. **Lexical Decision Task**

In the lexical decision task, participants were presented with a fixation cross (for 500ms), followed by a blank screen (for 1000ms), and a goal-congruent word (e.g., FRUIT) that was scrambled in half of the trials (e.g., TUIRF). In addition, we also presented participants with (scrambled) neutral (e.g., DRAAD, which means thread in Dutch, or RADAD when scrambled) and goal-incongruent words (e.g., SNOEP, which means candy in Dutch, or SEPON when scrambled). Participants had to indicate as fast as possible (but at least within 4000ms) whether the letters on the screen formed a word or a non-word. After each response, participants would be presented with their reaction time (RT). If no response was given, participants would receive feedback that they responded too slow.

Crucially, in half of the trials, participants were primed with the context they had chosen for goal-congruent behavior performance (e.g., lunch) for 50ms before target onset. This should render the goal (e.g., eating fruit) more accessible, especially for participants with stronger habits. As a consequence, the detection of goal-congruent words as words should be facilitated. To analyze this facilitation effect, we first calculated the priming effect by subtracting participants’ RT’s on non-prime trials from participants’ RT’s on primed trials. These difference scores were then subjected to a multilevel analysis, which showed no support for a priming effect (*t* = .18, *p* = .86), nor for an effect of time (*t­_linear_* = 1.60, *p* = .11; *t_quadratic_* = -.99, *p* = ..32).
